# Insights Into the Impact of Online Physician Reviews on Patients’ Decision Making: Randomized Experiment

**DOI:** 10.2196/jmir.3991

**Published:** 2015-04-09

**Authors:** Sonja Grabner-Kräuter, Martin KJ Waiguny

**Affiliations:** ^1^Department of Marketing and International ManagementAlpen-Adria-Universität KlagenfurtKlagenfurtAustria; ^2^Marketing, Advertising, Retailing and SalesAuckland University of TechnologyAucklandNew Zealand

**Keywords:** physician reviews, physician-rating website, physician choice making, patient experiences, word of mouth

## Abstract

**Background:**

Physician-rating websites combine public reporting with social networking and offer an attractive means by which users can provide feedback on their physician and obtain information about other patients’ satisfaction and experiences. However, research on how users evaluate information on these portals is still scarce and only little knowledge is available about the potential influence of physician reviews on a patient’s choice.

**Objective:**

Starting from the perspective of prospective patients, this paper sets out to explore how certain characteristics of physician reviews affect the evaluation of the review and users’ attitudes toward the rated physician. We propose a model that relates review style and review number to constructs of review acceptance and check it with a Web-based experiment.

**Methods:**

We employed a randomized 2x2 between-subject, factorial experiment manipulating the style of a physician review (factual vs emotional) and the number of reviews for a certain physician (low vs high) to test our hypotheses. A total of 168 participants were presented with a Web-based questionnaire containing a short description of a dentist search scenario and the manipulated reviews for a fictitious dental physician. To investigate the proposed hypotheses, we carried out moderated regression analyses and a moderated mediation analysis using the PROCESS macro 2.11 for SPSS version 22.

**Results:**

Our analyses indicated that a higher number of reviews resulted in a more positive attitude toward the rated physician. The results of the regression model for attitude toward the physician suggest a positive main effect of the number of reviews (mean [low] 3.73, standard error [SE] 0.13, mean [high] 4.15, SE 0.13). We also observed an interaction effect with the style of the review—if the physician received only a few reviews, fact-oriented reviews (mean 4.09, SE 0.19) induced a more favorable attitude toward the physician compared to emotional reviews (mean 3.44, SE 0.19), but there was no such effect when the physician received many reviews. Furthermore, we found that review style also affected the perceived expertise of the reviewer. Fact-oriented reviews (mean 3.90, SE 0.13) lead to a higher perception of reviewer expertise compared to emotional reviews (mean 3.19, SE 0.13). However, this did not transfer to the attitude toward the physician. A similar effect of review style and number on the perceived credibility of the review was observed. While no differences between emotional and factual style were found if the physician received many reviews, a low number of reviews received lead to a significant difference in the perceived credibility, indicating that emotional reviews were rated less positively (mean 3.52, SE 0.18) compared to fact-oriented reviews (mean 4.15, SE 0.17). Our analyses also showed that perceived credibility of the review fully mediated the observed interaction effect on attitude toward the physician.

**Conclusions:**

Physician-rating websites are an interesting new source of information about the quality of health care from the patient’s perspective. This paper makes a unique contribution to an understudied area of research by providing some insights into how people evaluate online reviews of individual doctors. Information attributes, such as review style and review number, have an impact on the evaluation of the review and on the patient’s attitude toward the rated doctor. Further research is necessary to improve our understanding of the influence of such rating sites on the patient's choice of a physician.

## Introduction

### Background

Online reviews are increasingly important sources of information for making different types of decisions. Recent industrial survey reports show that 90% of online shoppers read online reviews, and more than 80% of Internet users believe that these reviews affect their purchase behavior [1]. A growing number of people also share health care experiences online or rate the quality of their health care provider on physician-rating websites (PRWs) [2]. Thus, online resources, including advice from peers, are becoming a reliable source of health information, although the numbers still lag behind the numbers for commercial information searches [3]. The structure and content of PRWs are similar to other online rating websites in categories such as travel, hotels, or restaurants. PRWs typically provide information about a physician’s address, phone number, office hours, and certifications. However, the most important feature of PRWs is their focus on the expression of patient opinion about, and satisfaction with, a physician’s performance, which is online and visible to everyone. PRWs discuss the physician’s standards by using user-generated data and reflect the physician’s quality from the patient’s point of view [4,5].

Although a growing number of Internet users are consulting online ratings or reviews of doctors or other health providers, we still know little about public physician-quality reporting and its impact on patient choice behavior [3,6]. Recent research in this field has investigated the number, distribution, and trend of evaluations on physician-rating websites [4,7]. In previously published studies, the percentages of rated physicians in the US varied from 16%—estimation for all physicians in the US in the period of 2005 to 2010 [3]—to 27%—analysis of 300 randomly selected Boston physicians on 33 US PRWs in 2009 [8]. A more recent analysis of physician ratings on the German PRW, jameda, found that 37% of all physicians in the German outpatient sector were rated on jameda in 2012 [9]. Findings regarding evaluation valence are quite consistent among different studies, showing that the vast majority of reviews are positive. For example, Lagu et al [8] reported that 88% of quantitative, and 89% of narrative, patient reviews were positive. Similarly, for the German PRW, jameda, it was shown that about 80% of all evaluations could be assigned to the two best rating categories (ie, *very good* or *good*) [9]. All in all, it seems that not only the number of PRW users have increased during the last years, but also the relevance of physician reviews for patients’ decision processes.

### Physician-Rating Websites and Patients’ Evaluations of the Physicians

One topic which remains underresearched is the influence of PRWs when evaluating options and choosing a physician. More specifically, there are no studies that investigate the perception and effectiveness of online physician ratings and assess the impact of PRWs under experimental conditions [7]. We are aware of only one very recent study that examined the effect of the complexity of a choice set on consumers’ choices of physicians in an experimental context and found that the quality of choice deteriorates as choice sets incorporate more options and more performance metrics [10]. Experiments allow researchers to estimate the causal effect of manipulable treatments to which experimental subjects are randomly allocated on a given outcome [11]—in the context of PRWs, for example, the effect of certain review characteristics on the patient’s attitude toward the rated physician. There is also a research gap related to the content and nature of narrative reviews because most research in PRWs has focused on numerical ratings [12]. Recent investigations found that people not only evaluate ratings, but also scrutinize the written comments in online reviews [13]. Compared to simple numerical ratings (ie, star ratings or percentages), narrative reviews provide more detailed information and, thus, might add additional value to the decision-making process. Indeed, patients can write narrative commentaries in free text form on the vast majority of English-language and German-language PRWs [4,12,14]. However, the impact of narrative reviews depends on review characteristics such as review style and argument quality [15]. Consequently, this paper addresses this research gap and reports on an experimental study of the effects of review and context characteristics on patients’ perceptions of the review, the reviewer, and the reviewed physician. In doing so, this paper contributes to a better understanding of the role of review and context characteristics in the evaluation of online physician reviews. More precisely, this study investigates how a more or less factually written online physician review, in conjunction with a low or high number of reviews for the rated physician, affects the attitude toward the rated physician, the perceived expertise and trustworthiness of the reviewer, and the perceived credibility of the review.

### Determinants of Information Evaluation on Physician-Rating Websites

In recent years, considerable research has been directed at a better understanding of the effects of online rating sites on different aspects of choice behavior [16-20]. In these studies, different measures to investigate the effectiveness of online reviews have been used. In a comprehensive literature analysis of the impact of electronic word-of-mouth communication on consumer behavior [21], attitude, purchase intention, and product choice were identified as the most commonly investigated outcomes, followed by perceived usefulness, trust, and credibility constructs. Aspects of trust and credibility also have been shown to play a crucial role in the evaluation of online health information [22-24], and it can be assumed that they are at least equally relevant in the evaluation of physician ratings and reviews. Therefore, in this study we have chosen attitude toward the rated physician, and components of the message and source credibility as process and outcome variables.

Building on this line of work, information evaluation is considered a crucial determinant of the adoption of PRWs. According to traditional communication theories, a recipient’s information evaluation results from an interaction of message characteristics—related to message content, comprising factors such as valence or information quality—source or context characteristics (eg, expertise, trustworthiness), and receiver characteristics (eg, previous experiences) [25]. From this range of factors that potentially determine the influence of peer reviews on people’s decision making, in this study we have selected review style and number of reviews. As the effects of review valence have been extensively investigated by other researchers—with equivocal findings, though (eg, the review and synthesis of online word-of-mouth studies [26])—and as the majority of physician reviews on PRWs are positive [7,8,27], for this study we concentrate on the effects of positive reviews.

### Review Style

A number of communication and persuasion researchers have investigated how various elements of messages, such as language intensity, style, and quality, influence message perceptions—see the overview by Eastin [23]. However, only a few studies have investigated the relevance of textual content and linguistic style in online reviews [15,17,26,28]. Using a dataset from the Amazon.com website and combining text mining with econometric techniques, researchers have demonstrated that the writing styles and language used in reviews determine both consumers’ perceptions of those messages and their product choices [29]. It can be assumed that the style and the way reviews build up arguments for or against the rated physician also have an impact on how individuals evaluate the credibility of a review and the rated physician [30].

Consumer behavior literature offers contradictory findings about the influence of review style on review acceptance [18]. A qualitative study found that people prefer thematically structured, precise reviews that focus on simply describing the facts and refrain from extensively narrating the feelings of the reviewer [31]. That study defined perceived factuality as the conciseness, standardization, and specificity of an online review. In a similar vein, high-quality reviews are often operationalized as the ones that include relevant, comprehensive, and accurate product-related information [21,32]. In this respect, narrative and emotional expressions might be seen as a sign of subjectivity and, thus, reduce the perceived value of a review [33]. On the other hand, electronic peer-to-peer communication can be very influential because much of the information is presented in a narrative form and the emotional aspects of these narratives can be particularly persuasive [34]. A recent study found that reviews with emotional-laden elements, such as expressive slang and humor, were perceived as more valuable, at least up to a point [28]. Looking in the area of physician ratings, a recent context analysis found that narrative reviews for physicians sampled on the Yelp website, and in four English-speaking countries, lead to more usefulness ratings compared to pure fact-based reviews about the physician [35]. These heterogeneous arguments and prior findings prompt the consideration of some moderating variables when researching the influence of review style on review effectiveness. We assume that the influence of review style might interact with context factors, as further discussed, which might change if a more factually written review is more effective than a more emotionally written one.

### Number of Reviews

We expect the volume of online reviews to be an indicator of the intensity of the underlying effect of peer-to-peer recommendations [36]. Previous theoretical and empirical research has found a positive relationship between the volume of word-of-mouth communication and product sales [37,38]. People tend to put more trust in the recommendations from large numbers of reviewers [39]. With a larger number of reviews, the reader might arrive at the conclusion that she/he can learn from, and more easily rely on, the positive experiences other people have made comments about [40]. In a very recent study on credibility assessment in online health forums, Lederman et al found crowd consensus to be a highly relevant criterion for the evaluation of experiential health information, as it provides a group opinion regarding the validity of an experiential statement [41]. Given the volume and dispersion of online information, recipients frequently use certain heuristics to evaluate messages [10,26]. In this vein, another basis for understanding the influence of review number and its interaction with review style on the evaluation of online reviews can be derived from dual-process theories [42,43]. From a dual-process perspective suggesting the co-occurrence of effortless and effortful processing modes in certain situations, the number of reviews for a physician can serve as a mental shortcut or heuristic cue that reduces the amount of time and cognitive effort needed to process the message [21,44]. A large number of reviews might act as a cue for crowd consensus that simplifies the decision process and leads to a less critical processing of the information content of a single review. In this way, a larger number of reviews for an individual doctor can enhance the value of narrative and emotional expressions. These reviews might otherwise be seen as a sign of subjectivity that can reduce the effectiveness of a review. Thus, we expect that with a growing number of reviews, the positive impact of factually written reviews on a recipient’s information evaluation and the attitude toward the reviewed physician will become less salient.

### Hypotheses

#### Hypothesis 1

The number of reviews for a physician moderates the impact of a factual review style on the attitude toward the reviewed physician. Specifically, the effect of a factual style on attitude toward the reviewed physician will be weaker if the number of reviews is high.

In addition to attitude toward the reviewed physician, we assume that the effects are processed via selected measures of source and message credibility as other relevant outcomes of review and context characteristics. Persuasion research outlines that evaluations of source and message credibility are dominant process variables that lead to attitude changes [45].

Receivers of eHealth information in general, and readers of online physician reviews in particular, ought to be able to assess the credibility of the source and the message as important steps to information processing [46]. Credibility is the believability of a source or message [47] and has emerged as a critical indicator of eHealth information quality [48]. The components of source credibility that have been commonly identified are trustworthiness and expertise [49]. Trustworthiness refers to an information provider's intention to tell the truth or give unbiased information, and expertise refers to the extent to which an information provider is perceived capable of making correct assertions. Message credibility typically examines how message or information characteristics influence perceptions of believability. Major factors influencing message credibility include message structure, content and language, and plausibility of arguments [25,47].

Usually, writers of reviews stay anonymous and it is difficult for readers to assess source credibility and to determine if the information they receive is trustworthy [50]. Credibility research on media suggests that limited knowledge of source competence causes respondents to seek message-inherent heuristic cues (eg, language style and review number) to evaluate the information [23,51]. Cues embedded in the review and its presentation become characteristics with which to evaluate the message validity. In the context of this study, we examine the impact of review style and review number on both perceived credibility of the source (ie, expertise and trustworthiness of the reviewer) and the message. It is expected that more factually written physician reviews will induce higher levels of perceived expertise and trustworthiness of the reviewer and will lead to higher review credibility, and these effects will be moderated by the number of reviews.

#### Hypothesis 2

The number of reviews for a physician moderates the impact of a factual review style on the perceived expertise of the reviewer. Specifically, the effect of a factual style on perceived expertise of the reviewer will be weaker if the number of reviews is high.

#### Hypothesis 3

The number of reviews for a physician moderates the impact of a factual review style on the perceived trustworthiness of the reviewer. Specifically, the effect of a factual style on perceived trustworthiness of the reviewer will be weaker if the number of reviews is high.

#### Hypothesis 4

The number of reviews for a physician moderates the impact of a factual review style on the perceived credibility of the review. Specifically, the effect of a factual style on perceived credibility of the review will be weaker if the number of reviews is high.

Already, early studies in credibility research on media [52] found that the “trustworthiness” of a source significantly affected both the acceptance of the presented material and changes in opinion and attitude [23]. In the consumer behavior literature, dimensions of trust have been described as key mediating variables leading to positive attitudinal or behavioral relationship outcomes [53,54]. Following these lines of research, we also investigated the mediating effects of the credibility variables on the attitudinal outcome variable and suggest the following hypothesis.

#### Hypothesis 5

Perceived expertise and trustworthiness of the reviewer, as well as perceived credibility of the review, will mediate the interaction effect of style and review number on ratings of attitude toward the rated physician.

**Figure 1:**
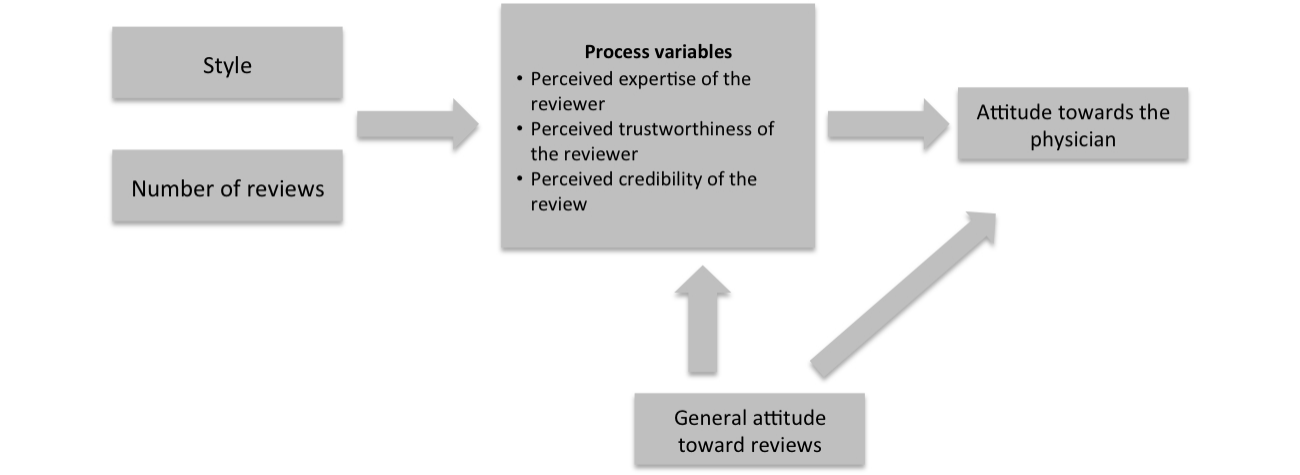
Influence of style and the number of reviews on attitude toward the reviewed physician.

Figure 1 summarizes the proposed model of how the style of the review and the number of reviews received influence attitude formation toward a physician.

## Methods

### Overview

To investigate the proposed hypotheses, we conducted a 2x2 online experiment—two review styles (emotional vs factual) by two numbers of reviews (low vs high)—between subjects, with 168 participants.

### Design

With careful isolation of the variables under consideration, the aim was to obtain an experimental design that allows for estimating the effects of review style and number. A search scenario for a dentist was chosen as a medium-to-high-involvement health service setting that seemed appropriate for a doctor choice that, in reality, could be primarily based on online reviews and would not allow for seeking personal word-of-mouth recommendations. Therefore, the scenario for an urgent need to consult a dentist because of a toothache shortly after relocation to a new city was created (see the description below). The choice of a general practitioner, for example, in the case of a flu, presumably could be based rather on practical decision criteria such as a short distance from the place of residence to the doctor’s office. On the other hand, in a situation where surgery is required it can be assumed that patients would not exclusively rely upon information provided on anonymous PRWs, but look for additional information from someone they personally know. Moreover, as both male and female participants were addressed, gynecologists and obstetricians were ruled out. On a final note, a search scenario for a dentist was considered more relevant for more people compared to, for instance, a search scenario for other specialists, such as orthopedists, internists, or pediatricians.

The doctor search scenario in the questionnaire was described as follows (in Austria, the term physician is used for dentists as well):

Please place yourself in the situation and imagine what this would be like for you:

You recently have moved to another city. As you suddenly have a racking toothache you start searching for a new dentist. Unfortunately, you do not know yet any dentist in this city and also cannot draw on recommendations from friends or acquaintances. Therefore, you decide to look for a dentist in your surroundings on the Web and read through online physician evaluations on this occasion.

You enter your search criteria on the Austrian physician rating website www.docfinder.at. On the next webpage, you are shown the profile of a dentist and the corresponding physician evaluation. Please read both descriptions carefully and then respond to the questions below.

The style of the reviews was manipulated by showing two versions of an online review for a fictitious dental practitioner on docfinder, the most popular PRW in Austria. The two review versions for the fictitious dentist, Dr Frank Weber, were created after a thorough and comprehensive analysis of the content, scope, and style of different reviews for dentists and other physicians on docfinder and other PRWs in Austria and Germany to keep them as realistic as possible. The reviews showcased a fictitious dentist visit and addressed the same order of topics in both versions. Moreover, care was taken to make sure the topics addressed in the two review versions could be assigned to some of the most relevant categories of patient concerns that have been identified in previous literature on narrative comments on PRWs, for example, as in Emmert et al [12]. One review was written in a more emotional way (eg, with the heading “fantastic doctor” and the use of emoticons such as a smiley, capital letters, and exclamation marks to strengthen the intensity of the positive message), while the other headline and the content were centered around facts about the physician’s service and the practice (eg, with the heading “modern technology and competent advice” and with only positive and concrete comments, no emoticons, no capital letters, and no exclamation marks). Both the fact-oriented and the emotional reviews included comments related to the professional competence, the efforts of the physician and the office staff, the practice equipment, and the waiting time to get an appointment. Friendliness of the physician and staff was not explicitly included in the fact-oriented review because it was considered to be more of an emotional attribute.

To check whether our manipulations worked, we asked how emotional or factual the review was perceived on a 7-point semantic differential, ranging from 1 (*emotional*) to 7 (*factual*). A *t* test for mean comparison showed a significant difference (*t*_*164*_=7.470, *P*<.001). Emotional reviews were rated as 2.79 (SD 1.32) while the factual reviews were rated as 4.47 (SD 1.55).

The number-of-reviews-received factor was manipulated by presenting two different versions of the physician profile for Dr. Frank Weber. Both versions of the profile included the physician’s address, telephone number, and the average overall rating of the physician—3 doctor’s cases out of 5 (*good* [73%])—in both versions. On docfinder, the average overall physician rating is shown with doctor’s case symbols (ie, icons that look like doctor’s medical bags). Ratings range from 0 doctor’s cases, meaning *insufficient*, to 5 doctor’s cases, meaning *excellent*. The manipulated review itself was rated as positive with 5 doctor’s cases out of 5. All this basic information was equal in both profile versions. Only the provided statistical information (ie, number of ratings and number of reviews received) was different in the two number-of-reviews-received conditions. The low number was 3 for both ratings and reviews received, while the high number was 30 for ratings and 27 for reviews received. Again, these numbers resulted from searching different physicians and noting how many reviews they received. On docfinder, in terms of review numbers, dentists considered the “top physicians” received about 10 times more reviews compared to the bottom-10%-rated physicians, who received the lowest number of reviews.

A 7-point scale assessing the perceived number of reviews, ranging from 1 (*few*) to 7 (*many*), served as a manipulation check. It showed that, generally, the 3 ratings and 3 reviews (low number) as well as the 30 ratings and 27 reviews (high number) were perceived as *few* to *average* number of reviews, but with a clear and significant difference between them (*t*_*164*_=8.889, *P*<.001). In the low number conditions, the mean perceived number of reviews was rated as 1.98 (SD 1.12), while the high number was rated as 3.87 (SD 1.64).

**Figure 2:**
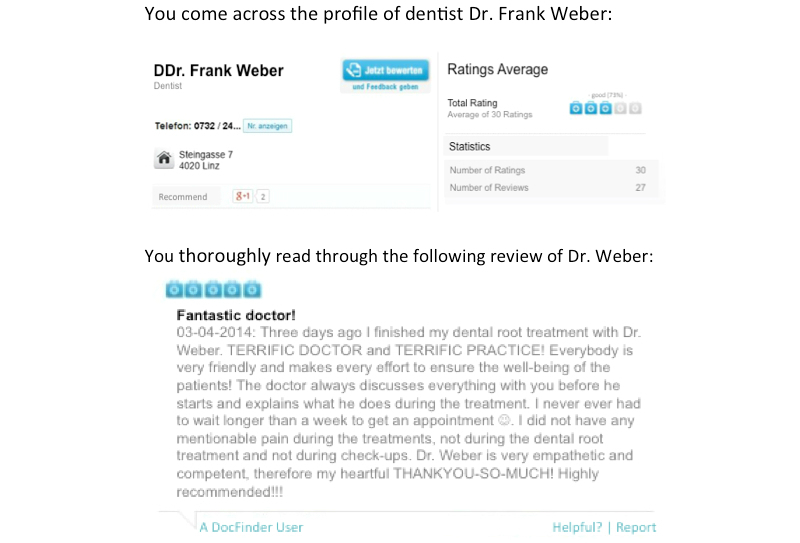
Manipulated rating card for the high-number/emotional review condition.

**Figure 3:**
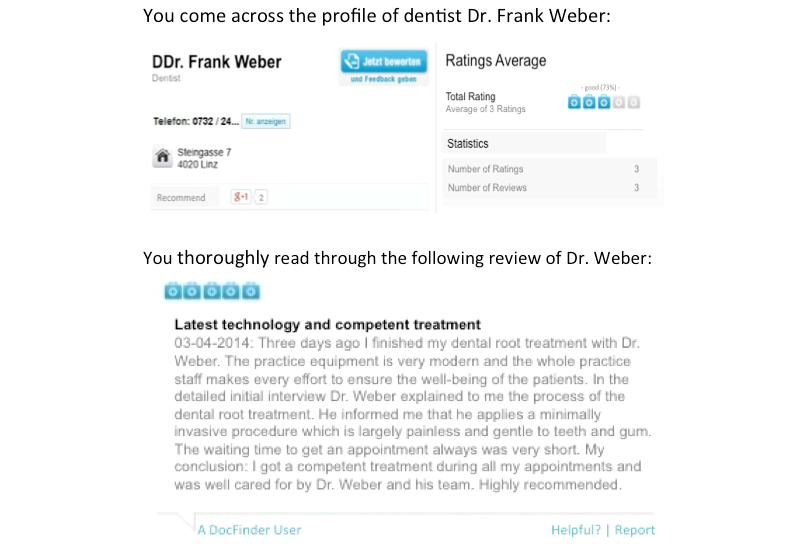
Manipulated rating card for the low-number/factual review condition.

In the following figures, the four experimental conditions are illustrated. The experimental report card of the physician profile above is for the manipulation of the number of received ratings (low vs high), while the review text below is for either the fact-oriented or emotional style manipulation. English translations of two report cards are provided in Figure 2 and Figure 3—the originals were in German.

### Measures

Established scales were used and adapted to a patient-doctor context, where needed, to measure each of the investigated constructs. The questionnaire was pretested on two experts and 10 other people in order to identify possible problems in terms of clarity and accuracy, and also to check to see if the created versions of the review and profile for the dentist were perceived as realistic. Thereafter, a few changes were made in order to improve the presentation order of the items, based on comments and feedback. Three items adapted from Hwang et al [55] gauged the attitude toward the physician using a 7-point scale (mean 3.94, SD 1.31, alpha=.942). Five items each, taken from Ohanian et al [56] assessed perceived expertise of the reviewer (mean 3.55, SD 1.26, alpha=.931) and perceived trustworthiness of the reviewer (mean 4.09, SD 1.29, alpha=.959), all of which were measured on 7-point agreement scales. Two items—the review is trustworthy and the review portrays a realistic picture of the physician—using a 7-point agreement scale [57], measured the perceived credibility of the review (mean 3.85, SD 1.25, alpha=.853). It can be assumed that any of the review-related perceptions of online reviews are influenced by how often people use reviews in general, and what attitude they have toward using them. Thus, we measured the general attitude toward online reviews (mean 4.37, SD 1.56, alpha=.934) with three items [58] and tested whether the slope direction was different between the groups. This check yielded no significant difference and, therefore, general attitude toward reviews was included as a covariate in the analyses to control for this influence. A PDF version of the questionnaire—German original, plus English translation—is provided in Multimedia Appendix 1.

### Sample

A total of 168 individuals clicked on the link to the online experiment and filled in the survey. Out of all the surveys, 2 cases were incomplete and could not be integrated in the analyses. Out of 166 participants, 50 (30.1%) were male. The average age was 27.7 years (SD 7.8), with a range from 16 to 58 years. Of 166 participants, 61 (36.7%) were full-time students, 60 (36.1%) were professionals, 35 (21.1%) reported that they were both studying and working, and 10 (6.0%) reported some other employment status. We checked whether age, gender, or occupation showed any significant differences among the randomized groups. Analysis of variance (ANOVA) and chi-square tests yielded no significant differences. Table 1 summarizes the distribution of occupation and gender among the randomized groups, as well as the average age of participants per experimental condition.

Only 7.2% (12/166) indicated that they never consult online reviews in general. However, many of the participants (75/166, 45.2%) have never written an online review, and 34.9% (58/166) indicate that they have written between one and three online reviews. Generally, the adoption of online reviews for health services compared to those for products and services is quite low, as 53.0% (88/166) of the participants have never consulted a physician-rating website before. Only 6.0% (10/166) have previously written a physician review.

**Table 1:** Characteristics of the sample (n=166).

Characteristics		Total, n (%) or mean (SD)	Experimental group, n (%) or mean (SD)			
			Low-number/ factual review (n=42)	Low-number/ emotional review (n=41)	High-number/ factual review (n=44)	High-number/ emotional review (n=39)
**Gender, n (%)**						
	Male	50 (30.1)	18 (43)	10 (24)	13 (30)	9 (23)
	Female	116 (69.9)	24 (57)	31 (76)	31 (70)	30 (77)
**Occupation, n (%)**						
	Student	61 (36.7)	17 (40)	17 (41)	17 (39)	10 (26)
	Professional	60 (36.1)	18 (43)	10 (24)	17 (39)	15 (38)
	Working and studying	35 (21.1)	5 (12)	12 (29)	8 (18)	10 (26)
	Other	10 (6.0)	2 (5)	2 (5)	2 (5)	4 (10)
Age in years, mean (SD)		27.7 (7.8)	27.1 (6.3)	28.4 (8.4)	28.1 (8.4)	27.0 (8.0)

### Procedure

The experiment was conducted online, using EFS Survey from Questback. The participants were recruited via email and social media. Undergraduate and graduate students at a mid-sized European university were invited to participate via an email message sent to their university email accounts. In addition, the link to the online survey was posted on the Facebook pages of the Department of Marketing and International Management and those of some of the research team members. Participants were also encouraged to actively forward the link to their friends.

**Figure 4:**
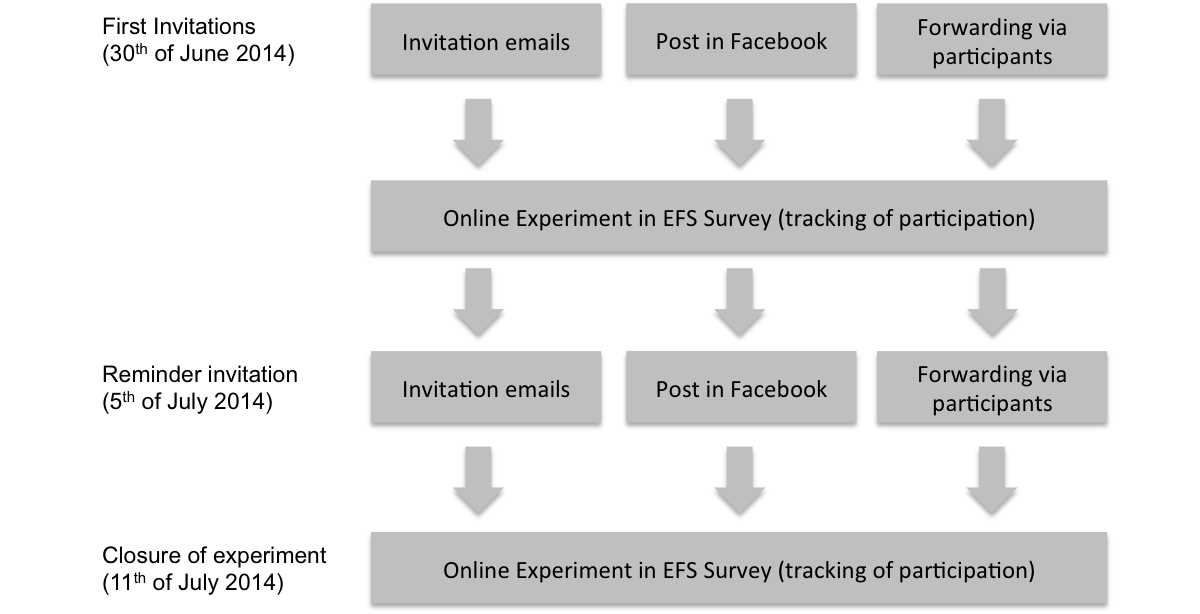
Recruitment process.

The questionnaire was available online from June 30 to July 11, 2014. In total, 264 participants opened the link to the experiment, 168 (63.6%) participants finished the survey, and 166 (62.9%) of all the surveys contained complete data on all measures (Figure 4).

First, respondents were asked some basic demographic questions. They were also asked about their use of online reviews in general, and online physician reviews in particular. They were then shown one of the four review conditions, which were randomly assigned by the software. Out of the 166 participants, 41 (24.7%) were exposed to the low-number-of-reviews/emotional condition, 42 (25.3%) to the low-number-of-reviews/factual condition, 39 (23.5%) to the high-number-of-reviews/emotional condition, and 44 (26.5%) to the high-number-of-reviews/factual condition. Right after participants read the review reports, we assessed the perceived credibility, followed by manipulation checks for review style, review number, and the attitude toward the dentist. Finally, attitudes toward review reports in general were recorded. After finishing the survey, if participants wanted, they could have their names entered into a raffle to win one of three €20 gift certificates.

To investigate the proposed hypotheses 1 to 4, we computed moderated regression analyses using the PROCESS macro 2.11 from Hayes for SPSS version 22 [59]. For testing the effects on the dependent variables, we applied the following settings: Model 1 (5000 bootstraps, confidence level of 95%), with the review style as the independent variable and the number of reviews as moderator, was estimated. The attitude toward online reviews in general was entered as a covariate to control for its influence in all of the analyses. Bootstrapping provides upper and lower level confidence intervals (ULCI and LLCI, respectively). If the range of these two does not include zero, the analysis shows significance. To investigate hypothesis 5, we calculated a moderated mediation model in PROCESS—Model 8: 2x2 design with two main effect variables and one interaction term—with perceived credibility of the review, perceived trustworthiness of the reviewer, and perceived expertise of the reviewer as mediators, and the two manipulated factors style and number as independent variables. Attitude toward the physician served as the outcome variable, with 5000 bootstraps and the three process variables—perceived expertise and trustworthiness of the reviewer, and perceived credibility of the review—as mediators. The number of reviews received, as well as the review style, were entered as independent variables, and general attitude toward reviews was entered as a covariate.

## Results

Hypothesis 1 proposed a positive effect of factual review style, which should be more prevalent if the physician received only a few reviews. The results of our regression model for attitude toward the physician suggest a positive main effect of the number of reviews (unstandardized regression coefficient [R]=0.398, *t*_*161*_=2.111, 95% CI 0.026-0.768, *P*=.04). Thus, when the dentist had more reviews, the attitude toward him was more positive (mean 4.15, standard error [SE] 0.13), compared to the condition where the dentist only had a few reviews (mean 3.73, SE 0.13). Besides this interesting main effect, the interaction effect of review style and number of reviews received was also significant (R=-0.798, *t*_*161*_=2.118, 95% CI -1.541 to -0.054, *P*=.04). As the effect was negative, this implies that the positive effect of a factual review style is mitigated when a high number of reviews is present.

**Figure 5:**
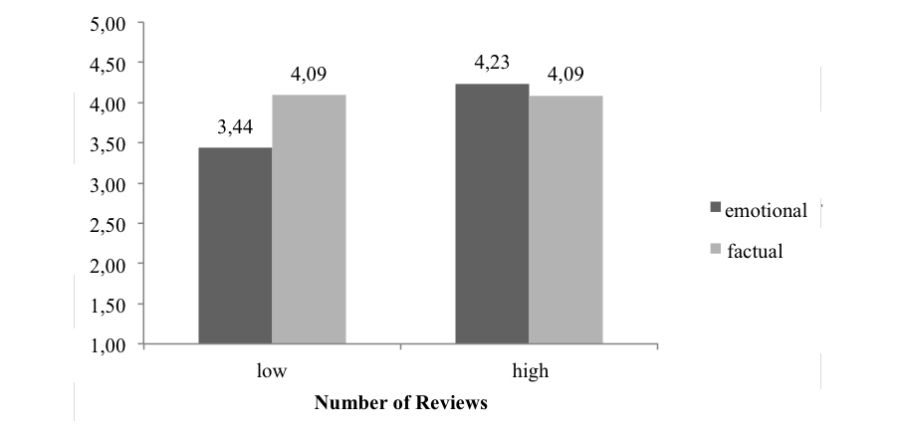
Attitude toward the physician as a function of review style and number of reviews.

We conducted single comparisons with conditional effect analyses, which revealed that the emotional review style lead to a less positive attitude (mean 3.44, SE 0.19) compared to the factual review style (mean 4.09, SE 0.19), if only a small number of reviews were received (95% CI 0.132-1.179, *P*=.01). If the physician received many reviews, factual and emotional review style did not result in different attitude ratings of the dentist. Furthermore, an emotional review style in the low-number-of-reviews-received condition also lead to a significantly less favorable attitude rating (mean 3.44, SE 0.19) compared to emotional reviews in the high-number-of-reviews-received condition (mean 4.23, SE 0.19) (95% CI 0.263-1.329, *P*=.004). The impact of the factual review style was not different in the two number-of-reviews-received groups. Figure 5 shows the interaction effect. In summary, factually written reviews performed better compared to emotional ones if the number of reviews received was low. However, if the physician had already received many reviews, the emotionally written review resulted in a more positive attitude. The covariate, general attitude toward online reviews, positively influenced the attitude toward the dentist (R=0.288, *t*_*161*_=4.861, 95% CI 0.179-0.423, *P*<.001). Thus, hypothesis 1 was confirmed.

In hypotheses 2 to 4, we predicted a positive effect of a factual review style on dimensions of source and message credibility. We also predicted that this effect would be moderated by the number of reviews received, as we expected that if only a few ratings and reviews were received, fact-oriented information would be more important. For each of the three variables, we calculated a separate moderated regression as we did for the test of hypothesis 1. The summaries of all these single regression models are displayed in Tables 2-4.

When testing hypothesis 2, we found a main effect of review style on perceived expertise of the reviewer. More factually written reviews (mean 3.90, SE 0.13) lead to a stronger perception that the reviewer had some expertise compared to the emotionally (mean 3.19, SE 0.13) written ones (R=0.710, *t*_*161*_=3.882, 95% CI 0.349-1.071, *P*<.001). All other effects were not significant, thus, hypothesis 2 is only supported for the main effect of review style (see Table 2).

When testing hypothesis 3 for the influence of review style and the number of reviews received on perceived trustworthiness of the reviewer, the moderated regression analyses yielded no significant effects, except for the covariate attitude toward reviews (R=0.207, *t*_*161*_=3.237, 95% CI 0.081-0.333, *P*<.001) (see Table 3). Thus, hypothesis 3 had to be rejected. However, the direction of the effect was similar to that for perceived credibility of the review. For the high-number-of-reviews-received condition, emotional and factual reviews lead to similar levels of perceived trustworthiness of the reviewer (mean 4.23, SE 0.20, mean 4.12, SE 0.19, respectively). However, for the low-number-of-reviews condition, the difference in the measure for perceived trustworthiness of the reviewer tended to be larger (mean 3.76, SE 0.20, mean 4.26, SE 0.20, respectively). Although not significant, the direction of the interaction effect did not contradict our other findings.

**Figure 6:**
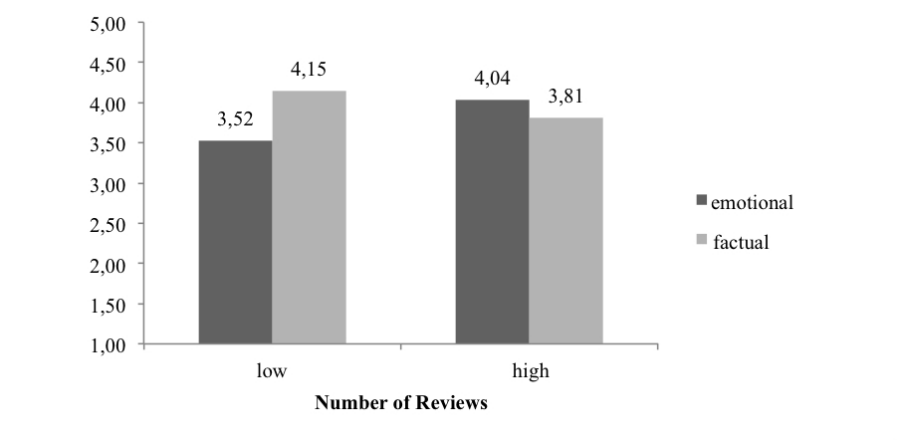
Credibility of the review as a function of review style and number of reviews.

When testing hypothesis 4 for the influence of review style and the number of reviews received on perceived credibility of the review, the moderated regression analysis confirmed the proposed interaction effect (R=-0.852, *t*_*161*_=-2.419, 95% CI -1.547 to -0.156, *P*=.02) (see Table 4). Single comparisons showed that in the condition where the physician received only a few reviews, factual reviews (mean 4.15, SE 0.17) were perceived as more trustworthy than emotional reviews (mean 3.52, SE 0.18, 95% CI 0.139-1.117, *P*=.01). Also, the comparison between emotional reviews in the low- and high-number-of-reviews-received conditions was significant. In the condition of many reviews received, emotionally written reviews resulted in higher credibility judgments (mean 4.04, SE 0.18) compared to emotional reviews in the condition of few reviews received (mean 3.52, SE 0.18, 95% CI 0.013-1.009, *P*=.045). Thus, hypothesis 4 is confirmed by our data. Figure 6 displays the interaction effect.

Hypothesis 5 proposes that the effects of review style and review number are mediated via the process variables perceived trustworthiness and expertise of the reviewer, as well as the perceived credibility of the review. Since we found significant main and interaction effects in the single analyses, we carried out a moderated mediation analysis as proposed by Hayes [59]. According to this analysis, it is essential that the effects from the independent variables on the mediators are established first. Furthermore, a relationship between the mediator and the dependent variable needs to be established—that is, a mediated indirect effect. Yet, nonmediated direct effects of the independent variables on the dependent variable are also possible. Thus, a mediation analysis must check to see if direct and indirect effects are observable [60]. To check this, the modeling tool PROCESS calculates a set of regression analyses. In the first two steps, the mediators and the dependent variables are regressed on the independent variables. Then, in a total model all independent variables (and possible covariates), as well as the mediator variables, are entered as predictors for the ultimate dependent variable. At the end, bootstrapping determines if the direct and indirect effects are significant. If both yield significance, we have partial mediation. If only indirect effects are significant, full mediation is observed, and if only direct effects are observed, no mediation occurs. To test the moderated mediation, bootstrapping is also applied. If the indirect effect of the highest order interaction bootstrapping results of LLCI and ULCI does not include zero in its range, a significant mediation effect is present.

Tables 2-5 summarize the results for the mediated regression analyses. The results for the regressions for the three mediators—hypotheses 2 to 4—showed a positive main effect of style on the perceived expertise of the reviewer, indicating that more factual reviews lead to higher expertise ratings. Furthermore, for credibility of the review, we found a significant interaction effect of review style and number of reviews received. The final total model includes all independent and mediation variables. The total effects of the independent variables, both indirect via the mediators and direct on the dependent variable, were calculated and displayed in Table 5. We followed the steps proposed by Zhao et al [59] to determine full or partial mediation and calculated the total regression model.

The total regression model showed a good predictive power (R^2^=0.62). When the mediators were entered into the model, attitude toward the physician was significantly influenced by the perceived trustworthiness of the reviewer (R=0.359, *t*_*161*_=5.101, 95% CI 0.220-0.498, *P*<.001), as well as by the perceived credibility of the review (R=0.430, *t*_*161*_=5.592, 95% CI 0.278-0.582, *P*<.001). Furthermore, the main effect of review number was also significant in the total model (R=0.281, *t*_*161*_=2.153, 95% CI 0.023-0.538, *P*=.03). The analyses confirmed that the influence of review style and number of reviews received was fully mediated via credibility of the review. This is seen as the direct effect becomes insignificant, and an indirect effect of the interaction term on the dependent variable, solely mediated via credibility of the review, is found (R of the indirect effect=-0.367, 95% CI -0.788 to -0.082, *P*=.05). Although we found a significant effect for the review style on the perceived expertise of the reviewer, the regression analyses showed that perceived expertise of the reviewer was not related to the attitude toward the physician, therefore, the main effect was not mediated. Thus, the proposed mediation effect in hypothesis 5 was only established for perceived credibility of the review, which fully mediated the interaction effect. General attitude toward reviews had a positive significant effect on all mediators, as well as on the dependent variable attitude toward the physician, indicating that people who are more positive toward reviews in general, show higher ratings in all outcomes.

In summary, the mediation analysis showed that perceived trustworthiness of the reviewer and credibility of the review influenced the attitude toward the doctor, as did the number of reviews received. However, the review style also had an influence. Our analysis suggests that factual reviews lead to a more positive credibility evaluation of the review, but only in the low-number-of-reviews-received condition. Due to the positive effect of review credibility on attitude toward the physician, the effect of style also transfers to the attitude toward the physician. The data partially support the hypothesis of a positive main effect of a fact-oriented review style. A more fact-oriented review style has a positive effect on the perceived expertise of the reviewer. However, perceived expertise of the reviewer was not a significant predictor for attitude toward the reviewed doctor. See Tables 2-5 for summaries of the above analyses.

**Table 2:** Results of the moderated mediation analyses for the outcome variable, perceived expertise of the reviewer.

Source^a^	R^b^	SE^c^	*t*	*P*	95% CI
Constant	2.798	0.278	10.070	<.001	2.249 to 3.347
Review style	0.710	0.183	3.882	<.001	0.349 to 1.071
Review number	0.289	0.182	1.586	.115	-0.071 to 0.648
Review style x review number	-0.293	0.365	-0.804	.422	-1.013 to 0.427
Attitude toward online reviews	0.169	0.060	2.826	.005	0.051 to 0.287

^a^Model summary: R=0.396, R^2^=0.157, F_4,161_=7.486, *P*<.001.

^b^Unstandardized regression coefficient (R).

^c^Standard error (SE).

**Table 3:** Results of the moderated mediation analyses for the outcome variable, perceived trustworthiness of the reviewer.

Source^a^	R^b^	SE^c^	*t*	*P*	95% CI
Constant	3.181	0.297	10.705	<.001	2.595 to 3.768
Review style	0.199	0.196	1.015	.312	-0.188 to 0.585
Review number	0.158	0.195	0.813	.417	-0.226 to 0.542
Review style x review number	-0.615	0.390	-1.577	.117	-1.385 to 0.155
Attitude toward online reviews	0.207	0.064	3.237	.002	0.081 to 0.333

^a^Model summary: R=0.294, R^2^=0.086, F_4,161_=3.800, *P*=.006.

^b^Unstandardized regression coefficient (R).

^c^Standard error (SE).

**Table 4:** Results of the moderated mediation analyses for the outcome variable, perceived credibility of the review.

Source^a^	R^b^	SE^c^	*t*	*P*	95% CI
Constant	2.520	0.268	9.390	<.001	1.990 to 3.050
Review style	0.202	0.177	1.144	.255	-0.147 to 0.551
Review number	0.085	0.176	0.484	.629	-0.262 to 0.432
Review style x review number	-0.852	0.352	-2.419	.017	-1.547 to -0.156
Attitude toward online reviews	0.309	0.058	5.353	<.001	0.195 to 0.422

^a^Model summary: R=0.429, R^2^=0.184, F_4,161_=9.085, *P*<.001.

^b^Unstandardized regression coefficient (R).

^c^Standard error (SE).

**Table 5:** Results of the moderated mediation analyses for the outcome variable, attitude toward the physician.

Source^a^	R^b^	SE^c^	*t*	*P*	95% CI
Constant	0.246	0.283	0.869	.386	-0.314 to 0.806
Expertise of the reviewer	0.079	0.059	1.329	.186	-0.038 to 0.196
Trustworthiness of the reviewer	0.359	0.070	5.101	<.001	0.220 to 0.498
Credibility of the review	0.430	0.077	5.592	<.001	0.278 to 0.582
Review style	0.043	0.136	0.314	.754	-0.226 to 0.311
Review number	0.281	0.130	2.153	.033	0.023 to 0.538
Review style x review number	-0.187	0.264	-0.710	.479	-0.708 to 0.334
Attitude toward online reviews	0.068	0.046	1.461	.146	-0.024 to 0.159

^a^Model summary: R=0.786, R^2^=0.617, F_7,158_=36.392, *P*<.001.

^b^Unstandardized regression coefficient (R).

^c^Standard error (SE).

## Discussion

### Principal Findings

The Internet has become an indispensable information medium for health services during the last years. Just as on other newly ubiquitous rating sites, an increasing number of people comment on their health care experiences online and rate the quality of their health care provider on physician-rating sites [2]. PRWs provide insight into the quality of care from the patient’s perspective and are a quick and easily accessible information source for patients who are seeking a physician. However, little is known about how physician reviews and ratings might affect physician choice making. As Schlesinger et al pointed out in a recent paper, consumer choice of doctors remains a “black box” that research has scarcely illuminated [10]. In their exploratory experimental study they investigate the impact of the complexity of information provided on PRWs on the quality of patients’ choice of doctors. Beyond that, with the growing use of PRWs, it is essential to identify which factors are most relevant for decision-making processes in this context [10]. One step in this direction is to improve our understanding of how patients develop attitudes toward doctors based on online reviews. Drawing on research from services as experience goods, we proposed a mediating impact of the expertise and trustworthiness of the reviewer, as well as the credibility of the review, on the attitude formation toward the physician. Furthermore, we expected that these relationships would depend on how the review is written and how many reviews the physician received.

We found that a more factual review style was positively related to the perceived expertise of the reviewer. This finding is in agreement with the findings of Lee and Koo [18] who showed that the credibility of online reviews with objective information was higher than that of online reviews with more emotional, subjective statements. The present findings also seem to be consistent with other research in the context of consumer products, which found that a more factual style is beneficial for the evaluation of the reviewer’s expertise and important for the generation of trust in the review [61]. Yet, in the current study the positive effect on perceived expertise as a dimension of source credibility did not translate into a more positive attitude toward the physician. And while trustworthiness of the reviewer positively influenced the attitude toward the physician, this effect was not dependent on the proposed independent factors investigated in our experiment. However, this study found that factual reviews lead to a more favorable attitude toward the physician only if a low number of reviews were received. We also found that the emotional reviews had greater impact when the physician received a high number of reviews. This interaction effect on attitude toward the physician was fully mediated by credibility of the review. Furthermore, we found a direct effect of the number of reviews received, which suggests that physicians were evaluated more favorably when they received more reviews. These findings corroborate previous research that found that physicians who received a higher number of ratings were shown to have better ratings [9].

### Implications

This leads to some important implications from the practitioner’s point of view. Firstly, it could be beneficial for physicians to obtain more reviews on their services. Similar suggestions from services marketing how to encourage patients to write reviews could be applied, like invitations or signage in the waiting area. Another approach to engage patients in writing reviews might work through the PRW itself. Similar to hotel booking services, if, for example, the contact to the physician is established via a PRW contacting option, the PRW could follow up with a reminder email to review the visit to the doctor.

Secondly, if only a few reviews are present and those are mainly emotional, even if they’re not negative, this leads to a less favorable attitude toward the physician and possibly reduces the probability of choosing this doctor. Yet, a physician could add some value here by responding to the reviews in a fact-oriented manner. This can easily be done if a feedback loop on PRWs is provided that allows physicians to respond to patients’ comments [12].

On the other hand, if a doctor received many reviews, a mix of emotional and factual reviews could be even more beneficial. This could be achieved by engaging different types of patients to write reviews. Also, these different types of reviews might attract further users of the PWR as the reviews might be perceived as more helpful if they contain different style elements. A recent content analysis on reviews for doctors on Yelp found that narrative reviews seemed to generate more usefulness ratings compared to short, pure fact-based ones [35].

Another minor finding from this study is the relatively low usage of health-related reviews compared to other product and service categories. While about 46% of our respondents reported that they have already consulted online reviews when searching for a physician, only 6% actually have written and posted a physician review themselves. These numbers are different from those reported by Emmert et al [7], who consulted a German online panel in January 2013 and found that only about 25% of all respondents had used a website when searching for a physician, but 11% had already posted a rating on a PRW. The differences could either be related to regional differences in the diffusion of PRWs between Germany and Austria or be a sampling effect because our experimental study relied on a convenience sample, as most experimental research does.

Taken together, the results of this research support the idea that more reviews are beneficial for a physician, as a high number of reviews might indicate her/his reputation as a sought-after practitioner. Our research also shows that people rate physicians more favorably as a result of both factual and emotional reviews if they have a wide array of reviews to choose from. However, in a situation of scarce information, more fact-oriented reviews lead to more favorable attitudes toward the reviewed physician.

### Limitations

There are some limitations to our study. First, we exposed the participants to only one physician review to see the effect of a more emotional or more fact-oriented review style, yet that did not necessarily reflect a real-world setting where comparisons and long lists of reviews are present. Moreover, in order to focus on the effect of review style and review number on attitude toward the rated physician and review and reviewer credibility, other characteristics of physician reviews, such as valence, length, review themes, and overall ratings, were controlled for. Future research could explore whether changes in these dimensions alter the effect of review style and number on review acceptance. Besides, as an online experiment was employed we could not control for distraction during the experiment. Another limitation is the exclusive focus on dentists. An investigation of the relevance of online reviews for the choice of other specialists or general physicians would not have to consider exactly the same topics or patients’ concerns, which also might have an effect on the influence of these reviews—future studies should address this point. Another limitation of our study is that we included only positive reviews. Therefore, testing the effect of review valence might be a valuable extension of the work. Finally, important limitations concern the nonprobability sampling technique and the narrowly focused online sample, which limited our ability to generalize the study’s findings to a broader population of patients. As the average age of our sample was only 27.7 years, the findings in particular provide some insights into how this younger age group evaluates online reviews of individual doctors. Further research might address the impact of age differences on the usage behavior of PRWs.

## Multimedia Appendix 1

Questionnaire used to measure investigated constructs—German original and English translation.

## Abbreviations

ANOVA: analysis of variance
PRW: physician-rating website
LLCI: lower level confidence interval
R: unstandardized regression coefficient
SE: standard error
ULCI: upper level confidence interval
