# Supplementary material for: Insights Into the Impact of Online Physician Reviews on Patients’ Decision Making: Randomized Experiment
Source: J Med Internet Res. 2015 Apr 9;17(4):e93. doi: 10.2196/jmir.3991 (PMC4408377; doi:10.2196/jmir.3991)
Supplement: Supplementary file 1 [file jmir_v17i4e93_app1.pdf]

Characteristics of online physician reviews: Some insights into their impact on patients' decision-making

APPENDIX - Survey

Sonja Grabner-Kräuter[1], Martin K.J. Waiguny[2]

[1] Associate Professor, Department of Marketing and International Management, Alpen-Adria-Universität Klagenfurt, Austria

[2] Senior Lecturer of Advertising, Auckland University of Technology, New Zealand

Address for correspondence:

Dr. Sonja Grabner-Kräuter

Dept. of Marketing and International Management

Alpen-Adria-Universität Klagenfurt

Universitätsstraße 56-56

9020 Klagenfurt, Austria

Phone: +43 463 2700 4042

Fax: +43 463 2700 994042

Email: Sonja.Grabner@aau.at

Fragebogen
Questionnaire

Liebe/r Teilnehmer/in,

herzlichen Dank für Ihr entgegengebrachtes Interesse und die Teilnahme an diesem Forschungsprojekt. Die Beantwortung der Fragen dauert etwa 10 Minuten. Alle erhobenen Daten werden anonym und vertraulich behandelt und ausschließlich für wissenschaftliche Forschungszwecke verwendet. Wer möchte, kann am Ende der Umfrage seine E-Mail Adresse hinterlassen und so die Chance nutzen, einen von drei Amazon Gutscheinen im Wert von je 20 Euro zu gewinnen. Die Gewinner werden nach Teilnahmeschluss per E-Mail verständigt.

Wir danken Ihnen für Ihre Teilnahme.

Dear participant,

Thank you very much for your interest and your participation in this research project. Answering the following questions will take about 10 minutes. All provided data are anonymous and strictly confidential and only will be used for scientific purposes. After finishing the survey, you may provide your e-mail address if you want to have the chance to have your name entered in a raffle to win one of three EUR 20,- gift certificates. Winners will be notified via e-mail after the survey is closed.

Thank you very much for your participation.

Demographische Angaben
Demographics

Bitte geben Sie Ihr Alter an:
__ Jahre

Bitte geben Sie Ihr Geschlecht an:
__ männlich, weiblich

Bitte verraten Sie uns, ob Sie sich derzeit in Ausbildung befinden oder einem Beruf nachgehen:
Student/in, berufstätig, beides, sonstiges: _____________

Please indicate your age:
__ years

Please indicate your gender:
__ male, female

Please tell us if you are currently involved in the educational process or if you are working:
__ student, working, both, other: _____________

Nutzungserfahrung des Teilnehmers
Usage experience of the participant

Wie weit trifft folgende Aussage auf Sie zu: Wenn ich ein Produkt kaufe oder eine Dienstleistung in Anspruch nehme, lese ich mir im Vorhinein in der Regel Bewertungen zu diesem Produkt bzw. dieser Dienstleistung durch:
(1 = trifft gar nicht zu, 7 = trifft voll zu)

How far does the following statement apply to you: When I buy a product or a service I usually read through reviews for this product or service before.
(1 = not at all, 7 = very much so)

Wie häufig haben Sie selbst bereits eine Bewertung verfasst?

- noch nie
- 1-3 Mal
- 4-10 Mal
- häufiger als 10 Mal

How often have you already written a review yourself?

- *never*
- *1-3 times*
- *4-10 times*
- *more than 10 times*

Wie häufig haben Sie online nach einem Arzt oder einer Ärztin gesucht?

- noch nie
- 1-3 Mal
- 4-10 Mal
- häufiger als 10 Mal

How often have you looked for a physician online?

- *never*
- *1-3 times*
- *4-10 times*
- *more than 10 times*

Wie oft haben Sie selbst online-Arztbewertungen zu Rate gezogen?

- noch nie
- 1-3 Mal
- 4-10 Mal
- häufiger als 10 Mal

How often have you consulted online physician reviews?

- *never*
- *1-3 times*
- *4-10 times*
- *more than 10 times*

Wie oft haben Sie selbst eine online-Arztbewertung verfasst?

- noch nie
- 1-3 Mal
- 4-10 Mal
- häufiger als 10 Mal

How often have you written an online physician review yourself?

- *never*
- *1-3 times*
- *4-10 times*
- *more than 10 times*

Welches der der folgenden Arztbewertungsportale kennen Sie?

- o www.docfinder.at
- o www.medicalreport.at
- o www.arztbewertung.net
- o ich kenne gar keines
- o Sonstiges: ___________________

Which of the physician rating websites below do you know?

- o *www.docfinder.at*
- o *www.medicalreport.at*
- o *www.arztbewertung.net*
- o *I don't know any of them*
- o *Other: ___________________*

Wie häufig nutzen Sie Arztbewertungsportale?

- o *regelmäßig*
- o *unregelmäßig*
- o *nie*

How often do you use physician rating websites?

- o *regularly*
- o *unregularly*
- o *never*

Szenario und manipulierte Screenshots
Scenario and manipulated screenshots

Bitte versetzen Sie sich in das folgend beschriebene Szenario:
Sie sind vor kurzem in eine andere Stadt gezogen. Da Sie plötzlich starke Zahnschmerzen haben, brauchen Sie dringend einen Zahnarzt. Leider kennen Sie in dieser Stadt noch keinen Zahnarzt und können auch nicht auf Empfehlungen von Freunden und Bekannten zurückgreifen. Sie beschließen daher, im Internet nach einem Zahnarzt in Ihrer Umgebung zu suchen und lesen sich bei dieser Gelegenheit online-Bewertungen durch.

Sie geben Ihre Suchkriterien in das österreichische Arztbewertungsportal www.docfinder.at ein. Auf der nächsten Seite erscheint Ihnen das Profil eines Arztes und die dazugehörige online-Bewertung. Lesen Sie beides bitte genau durch und beantworten in weiterer Folge die gestellten Fragen.

Please place yourself in the situation and imagine what this would be like for you:
You recently have moved to another city. As you suddenly have a racking toothache you start searching for a new dentist. Unfortunately, you do not know yet any dentist in this city and also cannot draw on recommendations from friends or acquaintances. Therefore you decide to look for a dentist in your surroundings on the web and read through online physician evaluations on this occasion.

You enter your search criteria on the Austrian physician rating website www.docfinder.at. On the next webpage, you are shown the profile of a dentist and the corresponding physician evaluation. Please read both descriptions carefully and then respond to the questions below.

Screenshot: www.docfinder.at

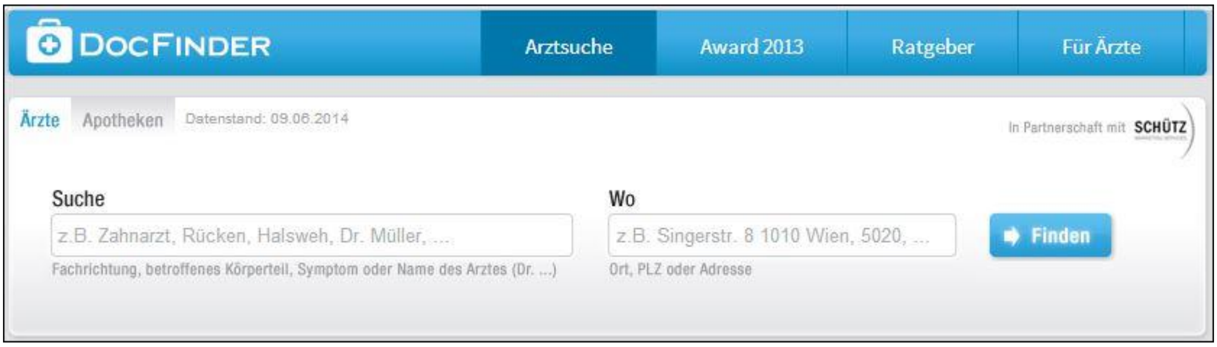

Random assignment of respondents to one of four experimental conditions through an automated randomization trigger provided by EFS Survey.

1. High number of ratings and reviews received – fact-oriented review

Sie stoßen auf das Arztprofil von Herrn DDr. Frank Weber:

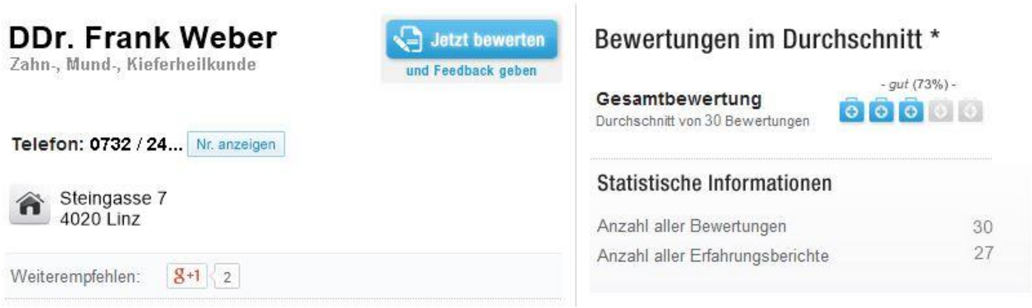

You come across the physician profile of Dr. Frank Weber:

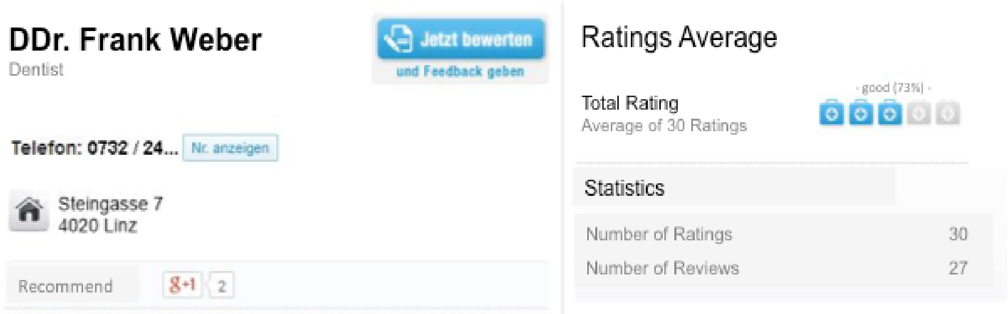

Folgende Bewertung von Herrn DDr. Weber lesen Sie sich genauer durch:

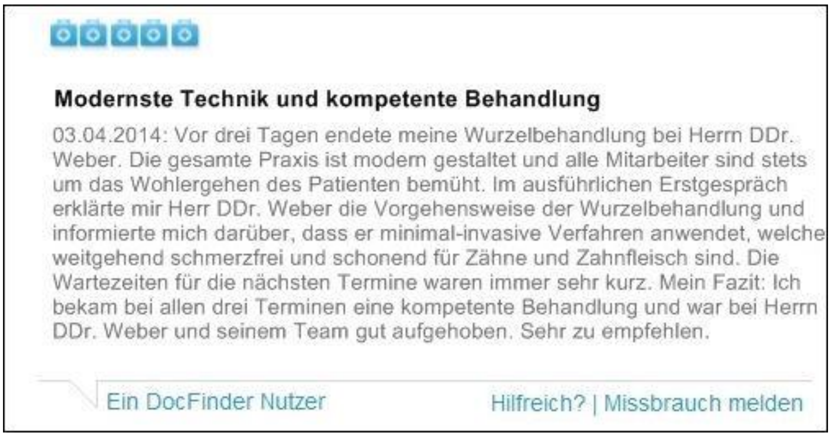

You thoroughly read through the following review of Dr. Weber:

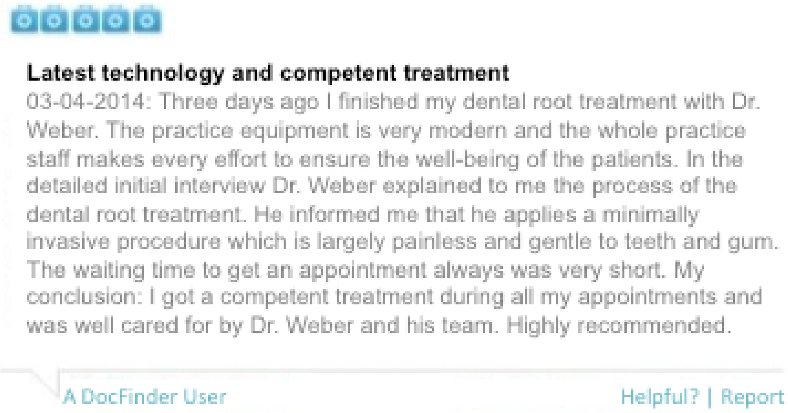

2. Low number of ratings and reviews received – fact-oriented review

Sie stoßen auf das Arztprofil von Herrn DDr. Frank Weber:

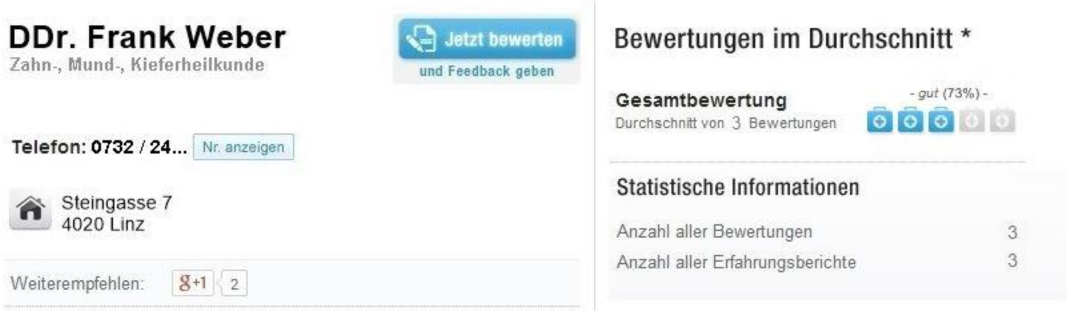

You come across the physician profile of Dr. Frank Weber:

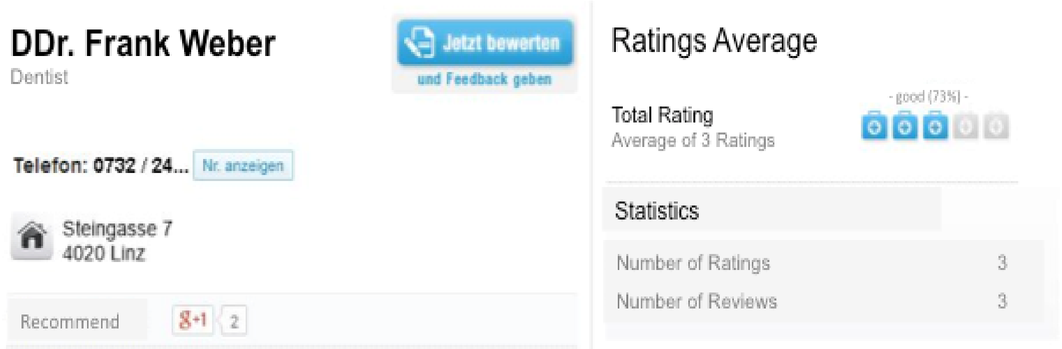

Folgende Bewertung von Herrn DDr. Weber lesen Sie sich genauer durch:

Modernste Technik und kompetente Behandlung

03.04.2014: Vor drei Tagen endete meine Wurzelbehandlung bei Herrn DDr. Weber. Die gesamte Praxis ist modern gestaltet und alle Mitarbeiter sind stets um das Wohlergehen des Patienten bemüht. Im ausführlichen Erstgespräch erklärte mir Herr DDr. Weber die Vorgehensweise der Wurzelbehandlung und informierte mich darüber, dass er minimal-invasive Verfahren anwendet, welche weitgehend schmerzfrei und schonend für Zähne und Zahnfleisch sind. Die Wartezeiten für die nächsten Termine waren immer sehr kurz. Mein Fazit: Ich bekam bei allen drei Terminen eine kompetente Behandlung und war bei Herrn DDr. Weber und seinem Team gut aufgehoben. Sehr zu empfehlen.

Ein DocFinder Nutzer | Hilfreich? | Missbrauch melden

You thoroughly read through the following review of Dr. Weber:

Latest technology and competent treatment

03-04-2014: Three days ago I finished my dental root treatment with Dr. Weber. The practice equipment is very modern and the whole practice staff makes every effort to ensure the well-being of the patients. In the detailed initial interview Dr. Weber explained to me the process of the dental root treatment. He informed me that he applies a minimally invasive procedure which is largely painless and gentle to teeth and gum. The waiting time to get an appointment always was very short. My conclusion: I got a competent treatment during all my appointments and was well cared for by Dr. Weber and his team. Highly recommended.

A DocFinder User | Helpful? | Report

3. High number of ratings and reviews received – emotional review

Sie stoßen auf das Arztprofil von Herrn DDr. Frank Weber:

DDr. Frank Weber
Zahn-, Mund-, Kieferheilkunde

Jetzt bewerten
und Feedback geben

Telefon: 0732 / 24... Nr. anzeigen

Steingasse 7
4020 Linz

Weiterempfehlen: +1 2

Bewertungen im Durchschnitt *

- gut (73%) -

Gesamtbewertung
Durchschnitt von 30 Bewertungen

Statistische Informationen

Anzahl aller Bewertungen	30
Anzahl aller Erfahrungsberichte	27

You come across the physician profile of Dr. Frank Weber:

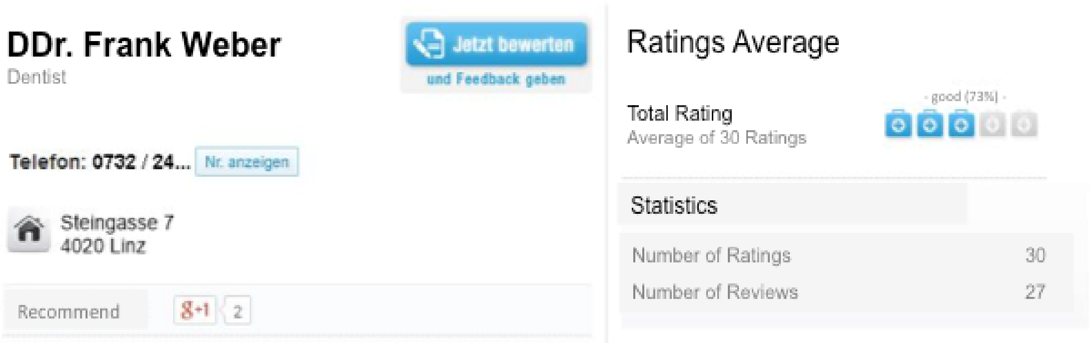

Folgende Bewertung von Herrn DDr. Weber lesen Sie sich genauer durch:

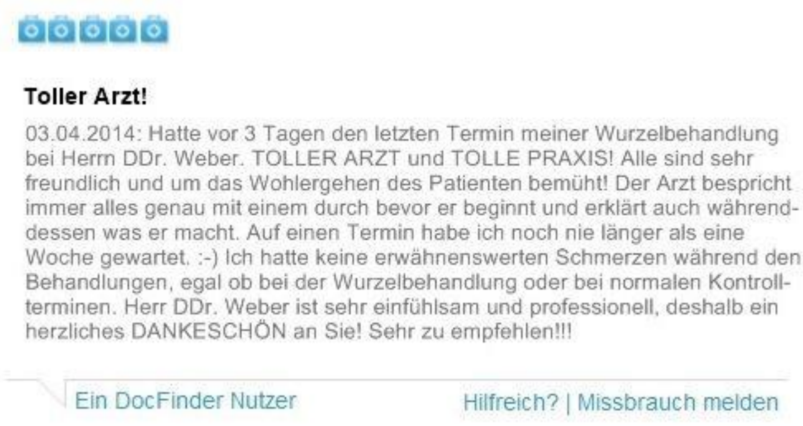

You thoroughly read through the following review of Dr. Weber:

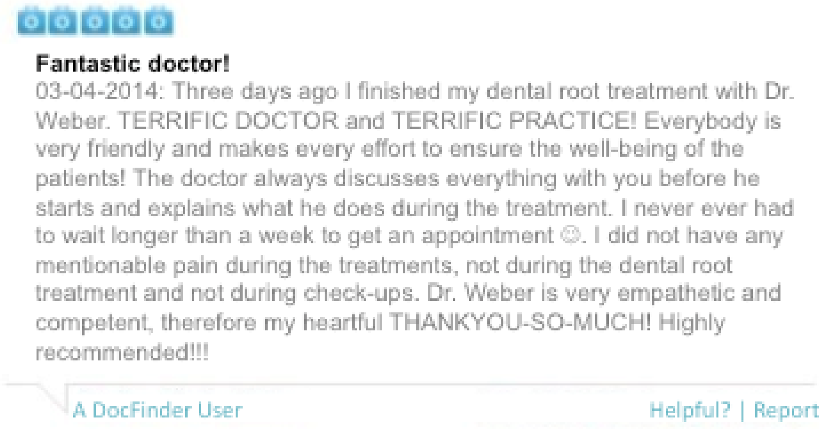

4. Low number of ratings and reviews received – emotional review

Sie stoßen auf das Arztprofil von Herrn DDr. Frank Weber:

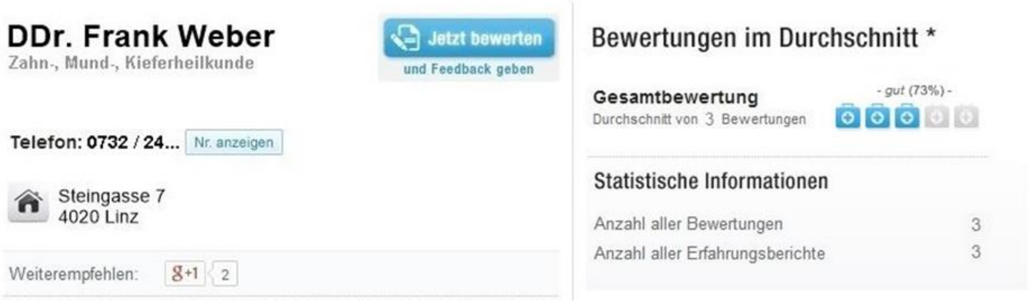

You come across the physician profile of Dr. Frank Weber:

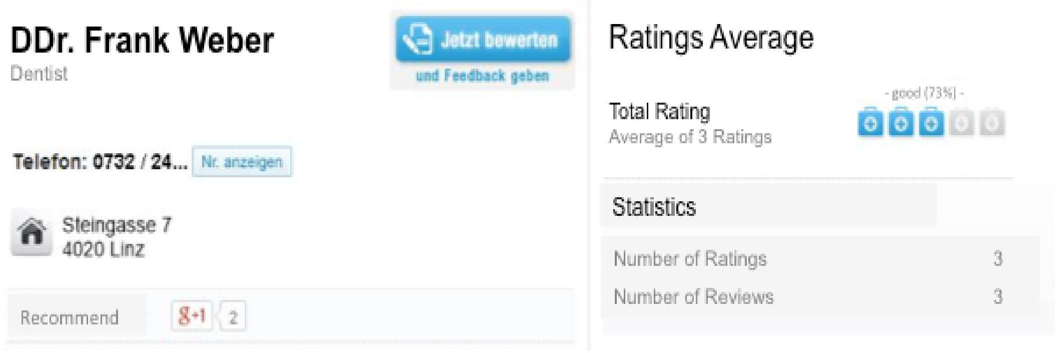

Folgende Bewertung von Herrn DDr. Weber lesen Sie sich genauer durch:

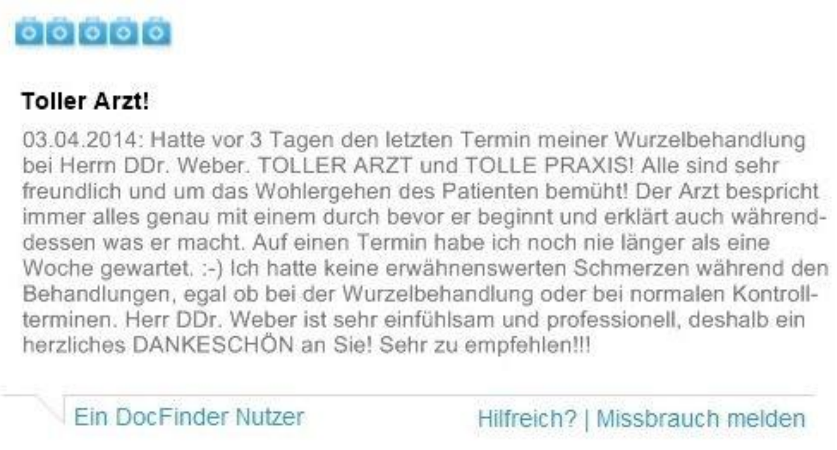

You thoroughly read through the following review of Dr. Weber:

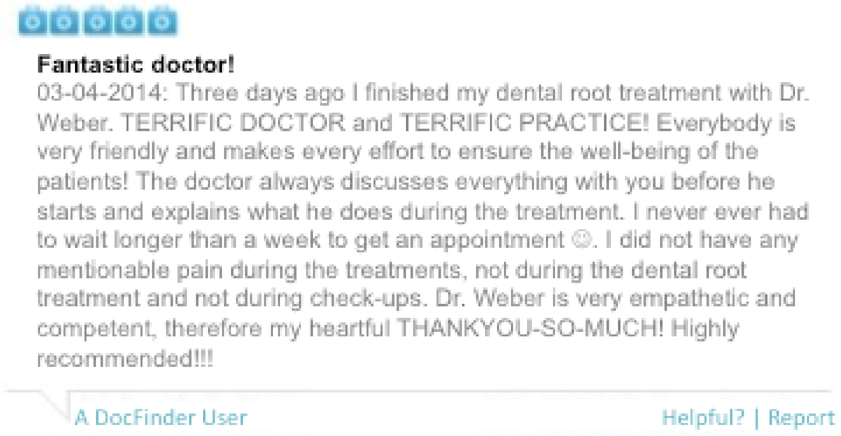

Fragen zur Beantwortung nach Präsentation der manipulierten Arztbewertung
Questions to be answered after reading one of the manipulated physician reviews

Wie beurteilen Sie die Vertrauenswürdigkeit der Arztbewertung, die Sie gerade gelesen haben?
(1 = gar nicht vertrauenswürdig, 7 = sehr vertrauenswürdig)

To what extent do you consider the review you just read as trustworthy?
(1 = not trustworthy at all, 7 = very trustworthy)

Inwieweit glauben Sie, dass die Bewertung, die Sie gerade gelesen haben, die Realität des Arztes wiederspiegelt?
1 (trifft gar nicht zu) – 7 (trifft voll zu)

To what extent do you consider the review reflects the reality of the physician?
(1 = not at all, 7 = very much so)

Wie beurteilen Sie die gelesene Bewertung?
(1= war emotional geschrieben, 7= war sachlich geschrieben)

How do you assess the review you just read?
(1= was written emotionally, 7 = was written fact-oriented)

Bitte beurteilen Sie die eben gelesene Bewertung anhand der folgenden Aussage:
Dieser Arzt wurde von vielen Personen bewertet.
(1 = trifft gar nicht zu, 7 = trifft voll zu)

Please assess the review you just read according to the following statement:
This doctor was evaluated by many people.
(1 = not at all, 7 = very much so)

Wie würden Sie den Verfasser der Bewertung einschätzen?
(7-teiliges Semantisches Differential)
nicht verlässlich – verlässlich
nicht ehrlich – ehrlich
nicht zuverlässig – zuverlässig
nicht aufrichtig – aufrichtig
nicht vertrauenswürdig – vertrauenswürdig
kein Experte – Experte
nicht erfahren – erfahren
nicht sachkundig – sachkundig
nicht qualifiziert – qualifiziert
nicht kompetent – kompetent

How would you assess the person who wrote the review?
(7-point Semantic differential)
undependable – dependable
dishonest – honest
unreliable – reliable
insincere – sincere
untrustworthy – trustworthy
not an expert - expert
unexperienced - experienced
unknowledgeable - knowledgeable

unqualified - qualified
unskilled - skilled

Bitte geben Sie an, wie Sie den Arzt, nach dem Lesen der online-Bewertung, beurteilen:
(1 = trifft gar nicht zu, 7 = trifft voll zu)
Dieser Arzt macht einen guten Eindruck auf mich.
Dieser Arzt überzeugt mich.
Ich wäre mit dem Arzt zufrieden.

Please indicate how you assess the doctor after having read the online review:
(1 = not at all, 7 = very much so)
This doctor makes a good impression.
This doctor convinces me.
I would be satisfied with the doctor.

Überlegen Sie bitte, ob folgende Aussagen auf Sie persönlich zutreffen oder nicht:
(1 = trifft gar nicht zu, 7 = trifft voll zu)
Meiner Meinung nach ist es lohnenswert, sich online-Bewertungen anderer Nutzer anzuschauen.
Ich glaube, es ist gut für mich, online-Bewertungen anderer Nutzer durchzulesen.
Alles in allem ist meine Einstellung gegenüber online-Bewertungen anderer Nutzer positiv.

Please consider if the following statements apply to you:
(1 = not at all, 7 = very much so)
In my opinion, it is worthwhile to read online reviews of other users.
I think it is good for me to read online reviews of other users.
Overall, my attitude towards online reviews of other users is favorable.

Gewinnspiel
Raffle

Ich möchte am Gewinnspiel teilnehmen und so die Chance auf den Gewinn eines Amazon Gutscheins im Wert von 20 Euro nutzen:

- o Ja.
  Bitte E-Mail Adresse eingeben: ____________________
- o Nein.

I would like to have my name entered into a raffle to be able to win one of three € 20,- gift certificates.
